# Three new species of genus *Onycholabis* Bates, 1873 (Coleoptera, Carabidae, Platynini) based on morphological and molecular data

**DOI:** 10.3897/zookeys.1282.186433

**Published:** 2026-06-16

**Authors:** Shokhrukh Bolkiboev, Sunbin Huang, Bakhtiyor Kholmatov, Hongbin Liang

**Affiliations:** 1 State Key Laboratory of Animal Biodiversity Conservation and Integrated Pest Management, Institute of Zoology, Chinese Academy of Sciences, Beijing 100101, China University of Chinese Academy of Sciences Beijing China; 2 University of Chinese Academy of Sciences, Beijing 100049, China State Key Laboratory of Animal Biodiversity Conservation and Integrated Pest Management, Institute of Zoology, Chinese Academy of Sciences Beijing China; 3 Institute of Zoology, Academy of Sciences of the Republic of Uzbekistan, Tashkent 100053, Uzbekistan Department of Entomology, College of Plant Protection, South China Agricultural University Guangzhou China; 4 Department of Entomology, College of Plant Protection, South China Agricultural University, 483 Wushan Road, 510642 Guangzhou, China Institute of Zoology, Academy of Sciences of the Republic of Uzbekistan Tashkent Uzbekistan

**Keywords:** *COI*, DNA barcode, genitalia, ground beetle, identification key, integrative taxonomy, morphology, taxonomy

## Abstract

Three new species of the genus *Onycholabis* Bates, 1873 are described, *O. hainanus* Bolkiboev & Liang, **sp. nov**. from Hainan; *O. tengjhihensis* Bolkiboev & Liang, **sp. nov**., and *O. cheni* Bolkiboev & Liang, **sp. nov**. from Taiwan, based on morphological comparisons and molecular analyses. Illustrations of habitus and genitalia with distribution maps are presented for the new species. A key to the nine known *Onycholabis* species is compiled, and a phylogenetic tree based on the barcode *COI* is provided.

## Introduction

The genus *Onycholabis* Bates, 1873 belongs to the tribe Platynini of the family Carabidae. Members of the genus are characterized by a glabrous body surface, slender whitish-yellow legs, elongated pubescent antennomere 3, and elongate, sharp mandibles. Adults are active at night, walking and predating along small streams, riverbanks, or splash zones of waterfalls.

Based on the works of Bates (1873, 1892), Andrewes (1923), Jedlička (1935, 1953), Louwerens (1955), Kasahara (1986, 1995), Paik and Lafer (1995), Liang and Imura (2003), Liang and Kavanaugh (2005) provided a brief revision recognizing a total of six species in the genus. Two decades have passed since then, and collecting efforts in China, including expeditions to Hainan and Taiwan, have yielded extensive specimens of *Onycholabis*, many of which were preserved for molecular analyses. Mr Changchin Chen also kindly presented us with numerous specimens collected from Taiwan. Our recent morphological comparison and molecular analysis have revealed three new species of *Onycholabis*, and their descriptions are given below.

## Materials and methods

This work is based on the examination of 172 specimens. All involved molecular samples, as well as GenBank accession numbers, are listed in Table 1. To ensure a stable rooting and verify the monophyly of *Onycholabis*, we selected outgroups with varying degrees of phylogenetic proximity: (1) closely related genera within the same tribe (Platynini), *Platynus* and *Agonum* (Liang and Kavanaugh 2005); (2) the more distant genus *Jujiroa* within the same tribe; (3) a representative from a different subfamily, *Dromius* (Lebiinae).

**Table 1.** *Onycholabis* and the outgroup used for the phylogenetic study with GenBank accession numbers.

Taxon	Voucher	Collecting data	*COI*
*Dromius angustus*	Outgroup	Outgroup	OQ716337
*Jujiroa zhouchaoi*	Outgroup	Outgroup	PX905444
*Platynus mannerheimii*	Outgroup	Outgroup	MZ607349
*Agonum piceolum*	Outgroup	Outgroup	PQ150013
*Onycholabis acutangulus*	GBony140	China: Yunnan, Gongshan, Cikai, 1510 m	PX905468
*O. cheni* sp. nov.	GBony008	China: Taiwan, Nantou, Luku, Shanlinxi (Sun Link Sea), 1350 m	PX905457
*O. cheni* sp. nov.	GBony009	China: Taiwan, Nantou, Luku, Shanlinxi (Sun Link Sea), 1600 m	PX905447
*O. cheni* sp. nov.	GBony010	China: Taiwan, Pingtung, Wutai, 1000 m	PX905460
*O. cheni* sp. nov.	GBony011	China: Taiwan, Pingtung, Machia, 1000 m	PX905458
*O. cheni* sp. nov.	GBony013	China: Taiwan, Keelung, Nuannuan, Laoliaokeng, 290 m	PX905449
*O. cheni* sp. nov.	GBony014	China: Taiwan, Keelung, Nuannuan, Laoliaokeng, 290 m	PX905463
*O. cheni* sp. nov.	GBony015	China: Taiwan, Keelung, Nuannuan, Laoliaokeng, 290 m	PX905455
*O. cheni* sp. nov.	GBony016	China: Taiwan, Keelung, Nuannuan, Laoliaokeng, 290 m	PX905459
*O. cheni* sp. nov.	GBony017	China: Taiwan, Keelung, Nuannuan, Laoliaokeng, 290 m	PX905453
*O. cheni* sp. nov.	GBony018	China: Taiwan, Keelung, Nuannuan, Laoliaokeng, 290 m	PX905445
*O. cheni* sp. nov.	GBony019	China: Taiwan, Keelung, Nuannuan, Laoliaokeng, 290 m	PX905452
*O. cheni* sp. nov.	GBony020	China: Taiwan, Keelung, Nuannuan, Laoliaokeng, 290 m	PX905450
*O. cheni* sp. nov.	GBony021	China: Taiwan, Keelung, Nuannuan, Laoliaokeng, 290 m	PX905446
*O. cheni* sp. nov.	GBony022	China: Taiwan, Keelung, Nuannuan, Laoliaokeng, 290 m	PX905451
*O. cheni* sp. nov.	GBony023	China: Taiwan, Keelung, Nuannuan, Laoliaokeng, 290 m	PX905464
*O. cheni* sp. nov.	GBony024	China: Taiwan, Keelung, Nuannuan, Laoliaokeng, 290 m	PX905454
*O. cheni* sp. nov.	GBony025	China: Taiwan, Keelung, Nuannuan, Laoliaokeng, 290 m	PX905456
*O. cheni* sp. nov.	GBony028	China: Taiwan, Kiayi, Chuchi Town, 1388 m	PX905461
*O. hainanus* sp. nov.	GBony026	China: Hainan, Baisha, Yuanmen, Yinggezui Station, 600 m	PX905465
*O. melitopus*	GBony147	China: Yunnan, Longling, Zhen-an Town, Mengliu	PX905467
*O. nakanei*	GBony145	Japan: Kanagawa, Tanzawa Mountains, Yadorigi-zawa	PX905469
*O. pendulangulus*	GBony106	Vietnam: Lao Cai, Lao Cai, Sa Pa, Ta Van, 997 m	PX905448
*O. pendulangulus*	GBony154	China: Yunnan, Tengchong, Wuhe Town, Longjiangqiao	PX905462
*O. sinensis*	GBony001	China: Fujian, Quangzhou, Dehua, Shangyong Houzhai	PX905443
*O. sinensis*	GBony004	China: Yunnan, Lvchun, Huanglianshan, 1790 m	PX905439
*O. sinensis*	GBony005	China: Yunnan, Lvchun, Huanglianshan, 1790 m	PX905441
*O. sinensis*	GBony029	China: Jiangsu, Lianyungang, Huaguoshan	PX905440
*O. sinensis*	GBony156	China: Yunnan, Jinping County, Adebo Town, Mazihe river	PX905442
*O. stenothorax*	GBony159	China: Yunnan, Longyang Bawan Town, Zhengding Station	PX905466
*O. tengjhihensis* sp. nov.	GBony006	China: Taiwan, Taitung, Peinan Town, Likia forest road, 1200 m	PX905437
*O. tengjhihensis* sp. nov.	GBony007	China: Taiwan, Pingtung, Taiwu, Peidawushan, 1200 m	PX905438

Collections cited in the present paper are indicated by the following abbreviations:

**CCCC** Collection of Changchin Chen, Tianjin, China

**IZCAS** Institute of Zoology, Chinese Academy of Sciences, Beijing, China

**TARI** Taiwan Agricultural Research Institute, Taichung, Taiwan

Measurements were taken using an ocular micrometer. Abbreviations for measurements used in the paper are as follows:

**BL** body length from apical margin of mandibles to elytral apex;

**HW** greatest width of head including eyes;

**EYL** eye length from anterior margin to posterior margin;

**TL** tempora length from posterior margin of eye to the narrowest point of neck;

**FW** width of the narrowest part of the frons between the eyes;

**PW** width of pronotum;

**PL** length of pronotum;

**PAW** width of pronotal apex;

**PBW** width of pronotal base;

**EW** width of elytra;

**EL** length of elytra (from the basal margin of the scutellum to the apex of the elytra);

**DAS** dorso-apical setae of all tarsomeres;

**LAS** latero-apical setae of all tarsomeres (Fig. 4).

Male genitalia were dissected and transferred to a cold 10% KOH solution for 8 h; the remaining membranes were removed under a compound microscope, and the cleaned genitalia were transferred to a drop of glycerin. The male reproductive tract was examined following the methods of Will et al. (2005). However, satisfactory results were obtained only from specimens preserved in approximately 75% EtOH, as material preserved in high-concentration EtOH or as dried-pinned specimens was unsuitable for examining soft internal tissues. As a result, detailed observations were limited to *Onycholabis sinensis* Bates, 1873 (Fig. 5). The terminology of male reproductive tract follows Will et al. (2005).

The entire abdomen of a female specimen was removed and transferred to a cold 10% KOH solution for 8–12 h. Following KOH treatment, abdomens were rinsed thoroughly with distilled water and dissected under a stereomicroscope. Dorsal tergites were gently separated from the sternites. The abdominal apex, including the female reproductive tract and hindgut, was stained with a saturated solution of Chlorazol Black® in methyl cellosolve for staining and enhanced visibility. In specimens with significant fat deposits around the cuticular structures, gentle tapping of the preparation allowed the solvent to dissolve remaining fat tissue, aiding in full structural clearance. Cleaned female genitalia were placed in a drop of glycerin in a plastic tube. The terminology of female genitalia follows Deuve (1993) and Liebherr and Will (1998).

For photography, the cleaned and relaxed male genitalia were placed in a drop of glycerin. Photographs of the habitus were taken with an Olympus camera model E-M1 mark II and M.Zuiko digital LED 60 f 2.8 Macro lens, afterward mounted on Helicon Focus (ver. 8). Photographs of male reproductive tract and tarsomeres were taken with Nikon camera model z 7 II and Phenix 4X (male reproductive tract) or 10X (tarsomeres) Plan achromatic objective, after mounted on Helicon Focus (ver. 8.3.7). Genitalia were photographed on a Keyence VHX-J00 digital microscope. Habitat images were taken using a Canon Mark III digital camera.

Total genomic DNA was extracted using the TIANamp Micro DNA Kit following the manufacturer’s protocols. To ensure successful amplification of the mitochondrial *COI* gene across diverse specimens, multiple primer sets were employed: the universal primers LCO1490 (5**'**-GGTCAACAAATCATAAAGATATTGG-3'), HCO2198 (5'-TAAACTTCAGGGTGACCAAAAAATCA-3') (Folmer et al. 1994); the degenerate primers Ill_B_F (5'-CCIGAYATRGCITTYCCICG-3'), Ill_C_R (5'-GGIGGRTAIACIGTTCAICC-3') (Lin et al. 2015); the modified primers M13F-mod (5'-TGTAAAACGAACGGCCAGTAAACTAATARCCTTCAAAG-3'), LepR1 (5'-TAAACTTCTGGATGTCCAAAA AATCA-3') (Hebert et al. 2004). PCR reactions were conducted in a 25 µL volume with annealing temperatures optimized at 50 °C. Amplicons were bi-directionally sequenced by Beijing Tianyi Huiyuan Biotechnology Co., Ltd.

Raw sequences were assembled in batches and manually verified using Geneious Prime (ver. 2025.0.2). For protein-coding sequences (CDS), alignments were performed using MACSE ver. 2.03 (Ranwez et al. 2018) to maintain the reading frame, followed by manual trimming of both ends. Phylogenetic reconstruction was carried out via IQ-TREE (ver. 3.0.1) with a codon-partitioning strategy (partitioned by 1^st^, 2^nd^, and 3^rd^ positions). The optimal substitution model for each partition was independently selected by ModelFinder Plus (MFP). To enhance the reliability of the topology, branch support was assessed using 1,000 replicates of both the SH-like approximate likelihood ratio test (SH-aLRT) and the Ultrafast Bootstrap (UFBoot), with the -bnni option enabled to reduce the risk of overestimating support due to severe model violations. For species delimitation, we employed Assemble Species by Automatic Partitioning (ASAP) via ASAPy (ver. 1.0) (Puillandre et al. 2021). This distance-based approach was used to infer putative operational taxonomic units (OTUs) and to complement the likelihood-based phylogenetic inference.

## Results

### Taxonomy

#### Onycholabis tengjhihensis

Taxon classificationAnimaliaColeopteraCarabidae

Bolkiboev & Liang sp. nov.

974A25E6-19E1-5996-B637-BEB7F6BC34DC

https://zoobank.org/5B7915D4-D7BA-41D5-8F4D-3FD9301CEA91

Fig. 1A

##### Chinese common name.

藤枝爪步甲.

**Figure 1.**
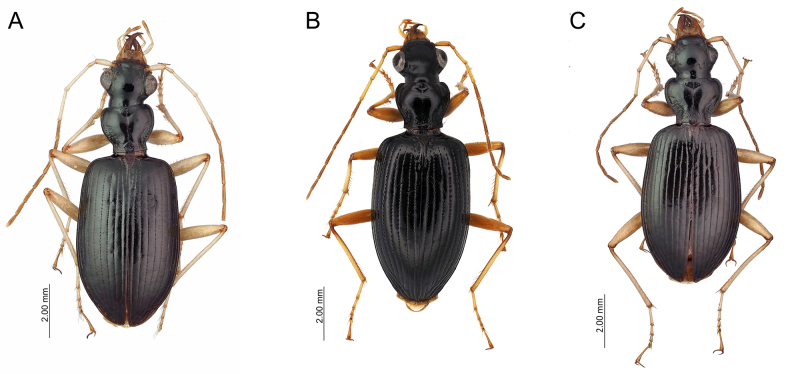
Holotype habitus of *Onycholabis* spp. **A**. *O. tengjhihensis* Bolkiboev & Liang, sp. nov.; **B**. *O. hainanus* Bolkiboev & Liang, sp. nov.; **C**. *O. cheni* Bolkiboev & Liang, sp. nov.

##### Type material.

***Holotype***. ♂ (TARI), China • Taiwan Province, Kaohsiung City, Taoyuan District, Baoshan Township, Tengjhih, 23.05667°N, 120.73917°E, 1220 m, 1996.08.08, leg. C.C. Chen, Holotype *Onycholabis tengjhihensis* det. Bolkiboev & Liang, 2026 [red label]. ***Paratypes***. total 4 ♀♀ and 3 ♂♂ (IZCAS and TARI) • 1 ♀, China, Taiwan Province, Ilan County, Tatung Township, 1998.04.11, leg. C.C. Chen • 1 ♀, China, Taiwan Province, Nantou County, Chushan Town, Shanlinxi, 2008.08.28, leg. Chen C.C. • 1 ♂, China, Taiwan Province, Taoyuan County, Fuhsing Township, Lalashan, 1994.5.8, leg. C.C. Chen • 1 ♂, China, Taiwan Province, Taitung County, Haituan Township, Wulu Village, 2008.10.22, leg. W.S. Lin • 1 ♂, China, Taiwan Province, Pingtung County, Taiwu Township, Peidawushan, 22.61417°N, 120.79361°E, 1200 m, 2022.08.25, leg. Y.T. Chung, C.C. Chen • 1 ♀, China, Taiwan Province, Taitung County, Peinan Township, Likia Forest Road, 1200 m, 2009.08.28., leg. W.I. Chou • 1 ♀, China, Taiwan Province, Hualien County, Hsiulin Township, Taroko National Park, 60–500 m, 2010.05.13, leg. W.I. Chou. Each paratype bears a yellow label: Paratype *Onycholabis tengjhihensis* det. Bolkiboev & Liang, 2026.

##### Description.

Length: 9.2–10.8 mm.

***Coloration***: Body black, shiny dorsally; mandibles, labrum reddish brown; antennomeres 1–4 pale yellow, others brown; maxillary palpi pale yellow, apical palpomere brown; legs yellow (tarsomeres 4 and 5, claws brown); ventral side reddish brown to black.

***Microsculpture***: Head hardly visible and formed of isodiametric meshes, pronotum and elytra formed of transverse meshes.

***Head*** with large and markedly prominent eyes (HW/FW = 1.74–1.78, mean 1.76, in four males and four females) and markedly constricted neck; tempora short, EYL/TL = 3.00–3.22 (3.15); vertex gently convex with surface very smooth and shiny, frontal furrows deeply concave, subparallel in front and slightly divergent posteriorly, anterolateral surface of frons longitudinally rugose; anterior supraorbital seta situated anterior to mid-eye level, posterior supraorbital seta situated slightly anterior to or on post-eye level; mandibles long, slender, gently curved inwards and gradually narrowing apically; apical maxillary palpomeres slender, shorter than penultimate ones, cylindrical in median portion and truncate at apices; apical labial palpomere shorter than the penultimate one; the latter one bi-setose; median tooth of mentum bifid, with each apex pointed; submentum 4-setose; antennae filiform, almost reaching the middle of elytra; relative lengths of scape and antennomeres II–XI as follows: 1.3 : 0.50 : 2.30 : 2.10 : 1.50 : 1.40 : 1.40 : 1.30 : 1.20 : 1.00 : 1.00.

***Pronotum*** cordate, wider than long, widest at about apical one-fifth, slightly narrower than head, PW/HW = 0.94–0.97 (0.95), PW/PL = 1.21–1.25 (1.22), PAW/PBW = 1.17–1.20 (1.18), PW/PAW = 1.30–1.33 (1.32), PW/PBW = 1.52–1.60 (1.57); apical margin widely and rather shallowly emarginate throughout; front angles rounded, obtuse at tips; lateral margins finely reflexed, arcuate before widest parts, rather gradually convergent to basal one-sixth, then strongly sinuate anterior to rectangular, pointed hind angles; single pair of marginal setae situated slightly before widest part; marginal setae at the hind angles are absent; basal margin distinctly bisinuate and unbordered throughout; disc moderately convex, with surface smooth, except for sparse and scattered large punctures along basal and lateral portions; median line deep and complete, basal foveae deep, rugose.

***Elytra*** subovate, moderately convex, widest slightly behind middle, much wider than pronotum, EL/EW = 1.58–1.61 (1.59), EW/PW = 1.9–2.0 (1.92); basal border not shortened, slightly curved; shoulders prominent and slightly rounded; lateral margins almost parallel-sided in basal portions, moderately convergent towards apices, subapical sinuation weak; apices conjointly rounded; scutellar striole long, punctate, not reaching interval 1; striae deeply impressed; intervals smooth, slightly convex in basal half and flatter towards apex; interval III with first discal pore at basal one-fifth near stria III and a second just behind middle and near stria II; interval IX with 17 umbilicate pores.

Prosternum sparsely punctate, proepisternum smooth, mesosternum smooth or sparsely punctate, mesoepisterna punctate, metasternum and metepisternum smooth; metepisterna long, 1.92 × as long as wide. Sternites 3–5 bi-setose; sixth visible sternite 4-setose in male, 8-setose in female.

***Legs*** long and slender; metacoxae bi-setose (inner seta absent); metafemora bi-setose; tarsi bisulcate; protarsomeres I–III with DAS and LAS, protarsi sulci shallow, metatarsomere I twice as long as metatarsomere II, metatarsomere IV bilobed, inner lobe equal to outer one, with LAS, metatarsomere V latero-ventrally asetose; claws simple (Fig. 4A–C).

**Figure 2.**
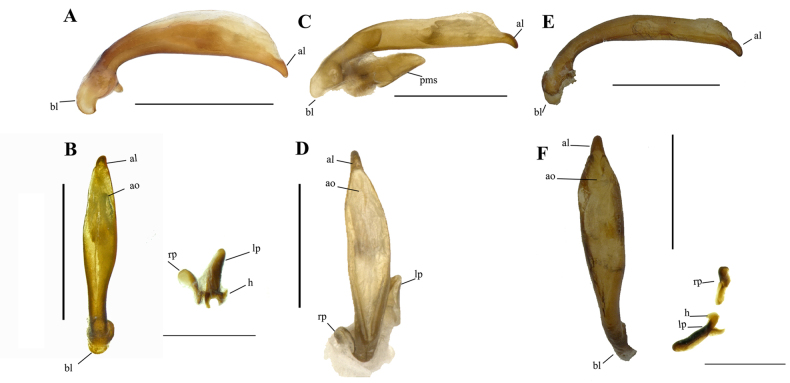
Holotype male genitalia of *Onycholabis* spp. **A**. *O. tengjhihensis* Bolkiboev & Liang, sp. nov.; **B**. *O. tengjhihensis* Bolkiboev & Liang, sp. nov.; **C**. *O. hainanus* Bolkiboev & Liang, sp. nov.; **D**. *O. hainanus* Bolkiboev & Liang, sp. nov.; **E**. *O. cheni* Bolkiboev & Liang, sp. nov.; **F**. *O. cheni* Bolkiboev & Liang, sp. nov. **A, C, E**. In lateral view; **B, D, F**. In dorsal view. Abbreviations: bl - basal lobe, al – apical lamella, ao – apical orifice; pms – parameres; lp – left paramere; rp – right paramere, h- hook (apophyse). Scale bar: 1.00 mm.

***Male genitalia***: Median lobe of aedeagus long and slender, moderately arcuate from basal lobe towards apex in lateral view; apical lamella sharply tapered towards the apex, 1.5 × longer than wide, narrowly rounded at the apex; apical orifice ending at two-thirds of median lobe and not reaching basal lobe. Left paramere broadly ovate and rounded at apex; right paramere small and narrow, with rounded apex. Endophallus not covered by tiny tooth-shaped sclerite scales; in lateral view, the endophallus with a broad, weakly convex distinctive fold in the middle section (Fig. 2A, B).

**Figure 3.**
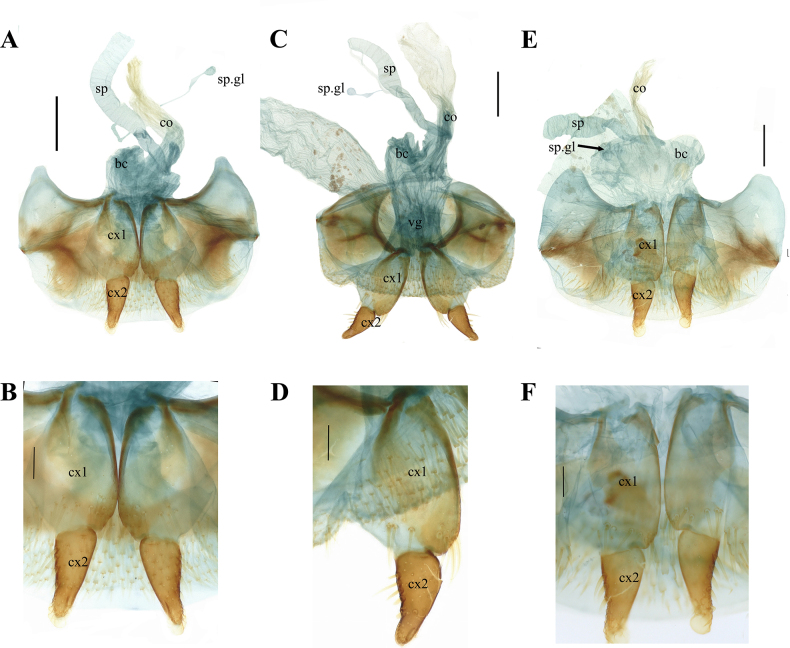
Female genitalia and reproductive tract of *Onycholabis* spp. **A, B**. *O. tengjhihensis* Bolkiboev & Liang, sp. nov.; **C, D**. *O. hainanus* Bolkiboev & Liang, sp. nov.; **E, F**. *O. cheni* Bolkiboev & Liang, sp. nov. Abbreviations: cx1 – basal gonocoxite; cx2 – apical gonocoxite; co – common oviduct; bc – bursa copulatrix; vg – vagina; sp – spermatheca; sp.gl – spermathecal gland. Scale bars: 0.5 mm (**A, C, E**); 0.2 mm (**B, D, F**).

***Female genitalia and reproductive tract*** as in Fig. 3A, B; apical gonocoxite > 1.5 × shorter than basal gonocoxite, slightly curved with a relatively sharp apex, apical gonocoxite with four dorsolateral and single dorsomedial ensiform setae; basal gonocoxite with apical fringe of 8–11 setae. Bursa copulatrix small, spermatheca long, strongly arcuate, C-shaped, evenly curved throughout, with short duct, and spermathecal gland with moderately large, spherical shape, and long duct.

**Figure 4.**
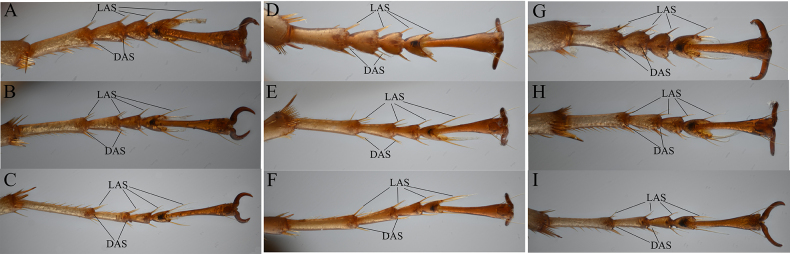
Tarsal morphology of the new *Onycholabis* species. **A, B, C**. *O. tengjhihensis* Bolkiboev & Liang, sp. nov. (female); **D, E, F**. *O. hainanus* Bolkiboev & Liang, sp. nov. (male); **G, H, I**. *O. cheni* Bolkiboev & Liang, sp. nov. (female). **A, D, G**. protarsi of *Onycholabis* spp.; **B, E, H**. mesotarsi of *Onycholabis* spp.; **C, F, I**. metatarsi of *Onycholabis* spp. Abbreviations: LAS – latero-apical setae; DAS – dorso-apical setae. Scale bar: 0.2 mm.

**Figure 5.**
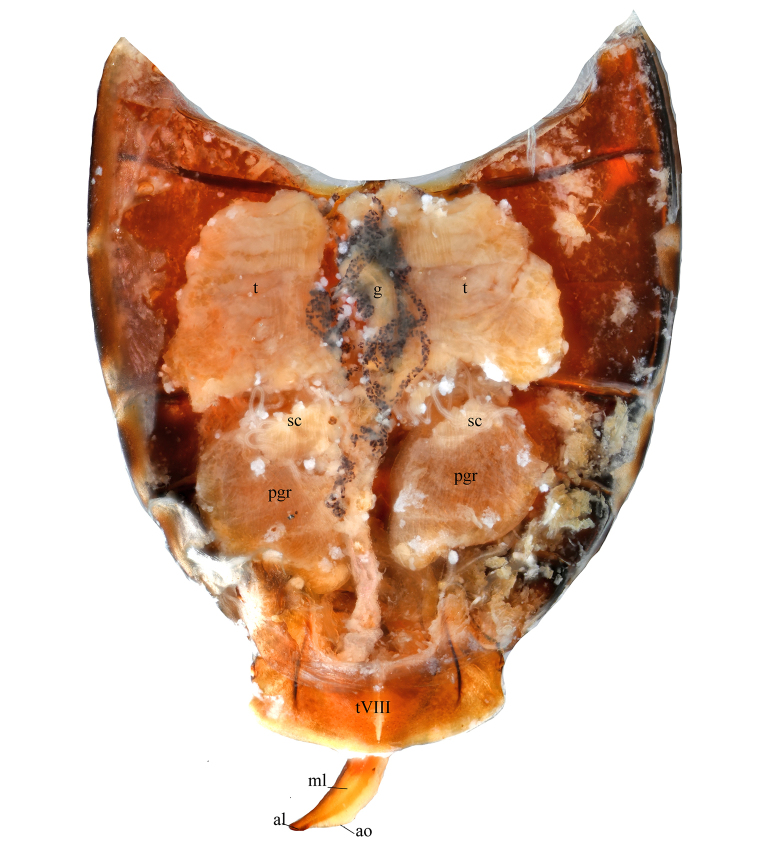
Male reproductive tract of *Onycholabis sinensis* Bates, 1873. Abbreviations: ep – epididymis; g – gut; pgr – pygidial gland reservoir; ml – median lobe of aedeagus; ao – apical orifice; al – apical lamella; sc – secretory cells; tVIII – tergite VIII; t – testis. Scale bar: 1.0 mm.

##### Differential diagnosis.

This new species is similar to *Onycholabis nakanei* Kasahara, 1986 in having the pronotum without posterior marginal setae, and the sixth visible sternites of males 4-setose, females 8-setose, but differs by tempora shorter, eyes > 3 × as long as tempora (eyes 1.77–2.08 × as long as tempora in *O. nakanei*); pronotum slightly narrower than head, PW/HW = 0.94–0.97 (PW/HW = 1.02–1.04 in *O. nakanei*); elytral interval III with two pores (three pores in *O. nakanei*); striae distinctly punctate (indistinctly punctate in *O. nakanei*). Also, the new species is closely related to *Onycholabis sinensis* Bates, 1873 in having large eyes, and the sixth visible sternite 4-setose in males; however, it differs in that the pronotum is narrower than head, PW/HW = 0.94–0.97, and without posterior marginal setae (pronotum wider than head PW/HW = 1.05–1.15 and with posterior marginal setae); elytral interval slightly convex (moderately convex in *O. sinensis*); median lobe of aedeagus moderately arcuate from basal lobe towards apex in lateral view (median lobe of aedeagus moderately arcuate at basal one-third, then almost straight toward apex in lateral view in *O. sinensis*).

##### Distribution.

China (Taiwan: Kaohsiung City, Ilan County, Nantou County, Taoyuan County; Taitung County; Pingtung County) (Fig. 8B).

##### Habitat.

One collector of this species, Mr Chang-Chin Chen, told us that he collected some specimens along the road and the others from the ground where he had set a light trap.

##### Etymology.

The new species name *tengjhihensis* refers to its type locality, Tengjhih, a famous forest recreation area in southern Taiwan.

#### Onycholabis hainanus

Taxon classificationAnimaliaColeopteraCarabidae

Bolkiboev & Liang sp. nov.

167F4875-47BF-5E46-9727-FB90160FF51A

https://zoobank.org/607C9929-DD79-4349-AB48-E63B6839692D

Fig. 1B

##### Chinese common name.

海南爪步甲.

##### Type material.

***Holotype***: ♂ (IZCAS), China, Hainan Province, Baisha County, Yuanmen Township, Yinggezui Station, 19.05208°N, 109.56480°E, 600 m, 2009.11.19, nighttime, leg. Hongbin Liang. Holotype *Onycholabis hainanus* det. Bolkiboev & Liang, 2026 [red label]. ***Paratypes***: 10 ♂♂ and 8 ♀♀ (IZCAS, TARI) • 8 ♂♂, 6 ♀♀, same data as holotype • 1 ♂, 1 ♀, same data as holotype but 2009.11.18 • 1 ♂, 1 ♀, China, Hainan Province, Lingshui County, Diaoluoshan, Xin-an, 18.72510°N, 109.86861°E, 921 m, 2012.04.16, daytime, leg. Hongliang Shi & Ye Liu. Each paratype bears a yellow label: Paratype *Onycholabis hainanus* det. Bolkiboev & Liang, 2026.

##### Description.

Length: 9.5–10.9 mm.

***Coloration***: Body black, shiny; mandibles, labrum reddish brown; antennomeres 1–4 pale yellow, others brown; maxillary palpi pale yellow, apical palpomere reddish yellow; femora brown, tibia pale yellow, tarsi pale yellow to brown; ventral side reddish brown to black.

***Microsculpture***: Head hardly visible and formed of isodiametric meshes, pronotum and elytra formed of transverse meshes.

***Head*** with large and markedly prominent eyes (HW/FW = 1.67–1.81, mean 1.74, in five males and five females) and markedly constricted neck; tempora short, EYL/TL = 3.00–3.44 (3.22); vertex moderately convex with surface very smooth and shiny; frontal furrows deeply concave, subparallel in front and slightly divergent posteriorly, anterolateral surface of frons longitudinally smooth; anterior supraorbital seta situated anterior to mid-eye level (in several specimens with two anterior supraorbital setae of left side), posterior supraorbital seta situated on post-eye level; mandibles long, slender, gently curved inwards and gradually narrowing apically; terminal maxillary palpomeres slender, shorter than penultimate palpomeres, cylindrical in median portion and truncate at apices; terminal labial palpomeres shorter than the penultimate; penultimate palpomeres bi-setose; median tooth of mentum bifid, with each apex sharply pointed; submentum 4-setose; antennae filiform, extending to the middle of elytra; relative lengths of scape and antennomeres II–XI as follows: 1.00 : 0.50 : 1.80 : 1.65 : 1.35 : 1.30 : 1.10 : 1.10 : 1.10 : 0.90 : 1.00.

***Pronotum*** cordate, wider than long, widest at about apical one-third, slightly narrower (in holotype) or as wide as head (in paratypes), PW/HW = 0.95–1.00 (0.98), PW/PL = 1.11–1.26 (1.18), PAW/PBW = 1.07–1.14 (1.10), PW/PAW = 1.25–1.35 (1.29), PW/PBW = 1.36–1.46 (1.42); apical margin widely and rather shallowly emarginate throughout; front angles rounded, obtuse at tips; lateral margins finely reflexed, arcuate before widest parts, rather gradually convergent to basal one-sixth, then slightly sinuate anterior to rectangular, pointed hind angles; single pair of marginal setae situated slightly before widest part, one pair of marginal setae situated at hind angle; basal margin distinctly bisinuate and unbordered throughout; disc moderately convex, with surface smooth, except for sparse and scattered large punctures along basal and lateral portions; median line deep and complete, basal foveae deep, rugose punctate.

***Elytra*** subovate, moderately convex, widest slightly behind middle, > 1.5 × wider than pronotum, EW/PW = 1.80–1.92 (1.87); EL/EW = 1.62–1.71 (1.67); basal border not shortened and slightly curved; shoulders prominent and slightly rounded; lateral margins almost parallel-sided in basal portions, moderately convergent towards apices, subapical sinuation weak; apices conjointly rounded; scutellar striole long, distinctly punctate and not joining interval I; striae deeply impressed, strongly punctate at basal half, weakly punctate in apical portion; intervals smooth, moderately convex in basal half, strongly convex in apical half and flatter towards apex; interval III with first discal pore at basal one-fifth near stria III and second one at the behind middle and near stria II, interval IX with 18 umbilicate pores.

Prosternum sparsely punctate or smooth, proepisternum punctate in inner part, mesosternum and mesoepisterna punctate, metasternum smooth or punctate laterally, metepisternum smooth; metepisterna long, 2.5 × as long as wide; visible sternites 3–5 bi-setose; sixth visible sternite bi-setose in male, 8-setose in female.

***Legs*** long and slender; metacoxae bi-setose (inner seta absent); metafemora bi-setose; tarsi bisulcate; protarsomeres I–III with DAS and LAS, protarsi sulci shallow, metatarsomere I almost 1.5 × as long as metatarsomere II, metatarsomere IV bilobed apically, inner lobe equal to outer lobe, with LAS, metatarsomere V latero-ventrally asetose; claws simple (Fig. 4D–F).

***Male genitalia***: Median lobe of aedeagus long and slender, slightly arcuate at basal one-third, then almost straight toward apex in lateral view; apical lamella rounded at apex in dorsal view, bent ventrally, twice as long as wide; apical orifice long, reaching basal lobe. Left paramere broadly ovate and rounded at apex; right paramere small and narrow, with rounded apex. Endophallus not covered by tiny tooth-shaped sclerite scales; in lateral view, endophallus with a shallow, elongate-ovate fold in the middle section and a distinct suboval sclerotized area near the basal third (Fig. 2C, D).

***Female genitalia and reproductive tract*** as in Fig. 3C, D; apical gonocoxite almost 2 × shorter than basal gonocoxite, slightly curved with a relatively sharp apex, apical gonocoxite with five dorsolateral and two dorsomedial ensiform setae; basal gonocoxite with apical fringe of 9–11 setae. Bursa copulatrix small, spermatheca long, moderately arcuate, C-shaped, evenly curved throughout, with short duct, and spermathecal gland with moderately large, spherical shape, and long duct.

##### Differential diagnosis.

This new species is closely related to *O. sinensis* Bates, 1873 in having the pronotum with two marginal setae, but differs by pronotum narrower than or as wide as head, PW/HW = 0.95–1.0 (pronotum wider than head, PW/HW = 1.05–1.15 in *O. sinensis*), sixth visible sternite bi-setose in male (4-setose in male of *O. sinensis*); apical lamella of the aedeagus comparatively longer and strongly bent ventrally (apical lamella of aedeagus shorter and not bent ventrally in *O. sinensis*).

##### Distribution.

China (Hainan: Baisha County; Lingshui County) (Fig. 8A).

##### Habitat.

Adults of the new species were found at night on stones near a shallow river (Fig. 9).

##### Etymology.

The new species name *hainanus* refers to its type locality, Hainan Province in southeastern China.

#### Onycholabis cheni

Taxon classificationAnimaliaColeopteraCarabidae

Bolkiboev & Liang sp. nov.

1A21F723-53CA-5250-886B-22CA9571176A

https://zoobank.org/285D44EF-5135-4A31-B561-50C873D7FA0D

Fig. 1C

##### Chinese common name.

陈氏爪步甲.

##### Type material.

***Holotype***. ♂ (TARI), China, Taiwan Province, Keelung City, Nuannuan District, Laoliaokeng, 25.0667°N, 121.7633°E, 290 m, 2020.05.16, nighttime, leg. S.P. Wu, Holotype *Onycholabis cheni* det. Bolkiboev & Liang, 2026 [red label]. ***Paratypes***. total 52 ♂♂ and 51 ♀♀ (IZCAS, TARI) • 7 ♂♂ and 4 ♀♀, same data as holotype, but 2020.03.28 • 1 ♂ and 1 ♀, same data as holotype, but 2020.04.11 • 2 ♀♀, same data as holotype, but 2020.05.16 • 6 ♂♂ and 3 ♀♀, same data as holotype, but 2019.04.18 • 3 ♂♂ and 6 ♀♀, same data as holotype, but 2020.01.29 • 2 ♂♂ and 1 ♀, same data as holotype, but 2020.02.07 • 7 ♂♂ and 3 ♀♀, same data as holotype, but 2020.02.26 • 3 ♂♂ and 1 ♀, same data as holotype, but 2020.02.29; 2 ♂♂ and 1 ♀, same data as holotype, but 2020.03.07 • 4 ♂♂ and 2 ♀♀, same data as holotype, but 2021.03.13 • 2 ♂♂ and 1 ♀, same data as holotype, but 2021.03.29 • 1 ♀, same data as holotype, but 2021.04.30, leg. S.P. Wu • 8 ♂♂ and 9 ♀♀, same data as holotype, but 2022.03.22 • 1 ♂, China, same data as holotype, but 2022.04.15 • 1 ♂ and 10 ♀♀, China, Taiwan Province, Chiayi County, Chuchi Township, Zhonghe branch of Alishan highway, 23.49075°N, 120.69969°E, 1388 m, 2017.09.21, nighttime, leg. Liang Hongbin • 3 ♀♀, same data, but 2017.09.22 • 1 ♂, China, Taiwan Province, Nantou County, Luku Township, Shanlinxi (Sun-link Sea Forest Recreation area), 23.6394°N, 120.7797°E, 1350 m, 2019.09.03, leg. B.H. Kuo, S.P. Wu & Y.T. Chung • 1 ♀, same data but 23.6375°N, 120.7844°E, 1600 m, 2019.09.09 • 2 ♂♂ and 1 ♀, China, Taiwan Province, Pingtung County, Wutai, 22.7539°N, 120.7828°E, 1000 m, 2019.10.04, leg. B.H. Kuo, & Y.T. Chung • 2 ♂♂, China, Taiwan Province, Pingtung County, Machia, 22.6736°N, 120.6761°E, 1000 m, 2019.10.04, leg. B.H. Kuo, & Y.T. Chung • 1 ♀, China, Taiwan Province, New Taipei City, Shihmen District, Laomei riverside, 1997.8.21, leg. S.P. Wu. Each paratype bears a yellow label: Paratype *Onycholabis cheni* det. Bolkiboev & Liang, 2026.

##### Description.

Length: 9.4–11.3 mm.

***Coloration***: Body black, shiny; mandibles, labrum reddish brown; antennomeres 1–4 pale yellow, others brown; maxillary palpi pale yellow, apical palpomere reddish yellow; femora brown, tibia pale yellow, tarsi pale yellow to brown; ventral side reddish brown to black.

***Microsculpture***: Head hardly visible and formed of isodiametric meshes, pronotum and elytra formed of transverse meshes.

***Head*** with small and slightly convex eyes (HW/FW = 1.58–1.82, mean 1.62, in five males and five females) and markedly constricted neck; tempora long, EYL/TL = 1.6–2.5, mean 2.20; vertex moderately convex with surface very smooth and shiny; frontal furrows deeply concave, subparallel in front and slightly divergent posteriorly, anterolateral surface of frons longitudinally smooth; anterior supraorbital seta situated anterior to mid-eye level (in several specimens with two supraorbital setae of left side, posterior supraorbital seta inserted on post-eye level); mandibles long, slender, gently curved inwards and gradually narrowing apically; terminal maxillary palpomeres slender, shorter than penultimate palpomeres, cylindrical in median portion and truncate at apices; terminal labial palpomere as long as the penultimate one; penultimate palpomeres bi-setose; median tooth of mentum bifid, with each apex sharply pointed; submentum 4-setose; antennae filiform, extending to the middle of elytra; relative lengths of scape and antennomeres II-XI as follows: 1.10: 0.60: 2.20: 1.90: 1.40: 1.40: 1.30: 1.10: 1.10: 1.00: 1.10.

***Pronotum*** cordate, wider than long, widest at about apical one-third, slightly narrower (in holotype) or as wide as head (in paratypes), PW/HW = 1.03–1.11 (1.07), PW/PL = 1.21–1.33 (1.26), PAW/PBW = 1.23–1.31 (1.26), PW/PAW = 1.25–1.35 (1.29), PW/PBW = 1.36–1.67 (1.42); apical margin widely and shallowly or moderately emarginate throughout; front angles rounded, obtuse at tips; lateral margins finely reflexed, arcuate before widest parts, rather gradually convergent to basal one-sixth, then slightly sinuate anterior to rectangular, moderately pointed hind angles; 1 pair of marginal setae inserted slightly before widest part, 1 pair of marginal setae situated at hind angle; basal margin distinctly bisinuate and unbordered throughout; disc moderately convex, with surface smooth, except for sparse and scattered large punctures along basal and lateral portions, median line distinctly impressed throughout, completed, basal foveae deep, rugose punctate.

***Elytra*** subovate, moderately convex, widest slightly behind middle, almost 2 × wider than pronotum, EW/PW = 1.76–1.92 (1.87), EL/EW = 1.61–1.71 (1.67); basal border not shortened and slightly curved; shoulders less prominent than in *O. hainanus* and moderately rounded; lateral margins almost parallel-sided in basal portions, moderately convergent towards apices, subapical sinuation weak; apices rounded and somewhat subtruncate at sutural angle; scutellar striole long, distinctly punctate and not joining interval I; striae finely impressed, finely punctate; intervals smooth, weakly convex; interval III with first discal pore at basal one-fifth near stria III and a second behind middle and near stria II, interval IX with 16 umbilicate pores.

Prosternum rugose, punctate, proepisternum punctate in inner part, mesosternum and mesoepisterna punctate, metasternum and metepisternum smooth; metepisterna long, 2.1 × as long as wide; sternites 3–5 bi-setose; sixth visible sternite bi-setose in male, 8-setose in female.

***Legs*** long and slender; metacoxae bi-setose (inner seta absent); metafemora bi-setose; tarsi bisulcate; protarsomeres I–III with DAS and LAS, protarsi sulci shallow, metatarsomere I > 1.5 × as long as metatarsomere II, metatarsomere IV bilobed apically, inner lobe slightly longer than outer lobe, with LAS, without LSAS, metatarsomere V latero-ventrally asetose; claws simple (Fig. 4G–I).

***Male genitalia***: Median lobe of aedeagus long and robust, slightly arcuate at basal one-third, then almost straight toward apex in lateral view, apical lamella rounded at apex in dorsal view, slightly bent ventrally; apical orifice ending at two-thirds of median lobe and not reaching basal lobe. Left paramere broadly subovate and rounded at apex; right paramere small and narrow, with rounded apex. Endophallus not covered by tiny tooth-shaped sclerite scales; in lateral view, endophallus with a weak, balloon-like, distinct fold in the middle section (Fig. 2E, F).

***Female genitalia and reproductive tract*** as in Fig. 3E, F; apical gonocoxite 1.5 × shorter than basal gonocoxite, slightly curved with a relatively sharp apex, apical gonocoxite with 3 or 4 dorsolateral and 1 dorsomedial ensiform setae; basal gonocoxite with apical fringe of 8–10 setae. Bursa copulatrix small, spermatheca long, moderately arcuate, C-shaped, evenly curved throughout, with short duct, and spermathecal gland with small, spherical shape, and long duct.

##### Differential diagnosis.

This new species is closely similar to *O. sinensis* Bates, 1873, in having the pronotum cordate and with two marginal setae but differs in having the sixth visible sternite bi-setose in male (4-setose in male of *O. sinensis*). It is also closely similar to *O. hainanus* Bolkiboev & Liang, sp. nov. in having the pronotum cordate and with two latero-marginal setae, but differs by the pronotum wider than head (PW/HW = 1.03–1.11) and moderately wider than long (PW/PL = 1.21–1.33), (pronotum narrow or as long as head (PW/HW = 0.95–1.0) and pronotum slightly wider than long (PW/PL = 1.11–1.26) in *O. hainanus*); elytra striae indistinctly punctate (elytra striae distinctly punctate in *O. hainanus*); median lobe of aedeagus robust and apical lamella slightly bent ventrally (median lobe of aedeagus slender and apical lamella strongly bent ventrally in *O. hainanus*).

##### Distribution.

China (Taiwan: Keelung City; Pingtung County; Nantou County; Chiayi County; New Taipei City) (Fig. 8B).

##### Habitat.

At night, adults were collected while climbing a vertical stone surface close to a small waterfall in Chiayi County on 21 September 2017, hiding under stones in the riverbed. According to the observations of collector, Dr Shu-Ping Wu, they were abundant from January to April in a stream in Laoliaokeng (Fig. 10).

##### Etymology.

The new species name, *cheni*, is named in honor of Mr Chen Chang-Chin (陈常卿), who contributed greatly to our Carabidae research.

### Phylogeny

A total of 32 sequences of the *COI* gene for *Onycholabis* were obtained, with lengths ranging from 480–861 bp (mean = 551 bp). After quality filtering, the final alignment included 36 sequences (32 in-group and 4 outgroup taxa). Maximum Likelihood phylogenetic inference was performed using IQ-TREE based on the dataset with outgroups. The best-fit nucleotide substitution models were determined for each partition (TN+F+G4, F81+F+I, and HKY+F+G4 for partitions 1, 2, and 3, respectively).

With the distant outgroup *Dromius* as the root, the tree topology robustly confirmed the close phylogenetic affinities among the studied groups. *Jujiroa* branched off first among the Platynini outgroups (SH-aLRT/UFBoot support: 87.7/83), followed by *Agonum* (82/53). *Platynus* was recovered as the sister group to the strongly supported monophyletic genus *Onycholabis* (90.8/61).

Within the ingroup, *Onycholabis* diverged into two main clades. The first main clade (79.3/69) comprised two sister lineages: one containing *O. pendulangulus* Liang&Imura, 2003 and *O. acutangulus* Andrewes, 1923 (89.2/96), and the other (98.1/98) recovering *O. nakanei* Kasahara, 1986 as sister to a subclade consisting of *O. sinensis* Bates, 1873 and *O. tengjhihensis* sp. nov. (79/92). The second main clade (23.5/14) grouped the remaining species. Within it, *O. melitopus* Bates, 1892 and *O. stenothorax* Liang & Kavanaugh, 2005 formed a strongly supported subclade (99.1/100) that was recovered as sister to *O. hainanus* sp. nov., though with weak support (43.9/41). This group was then recovered as sister to the highly supported monophyletic clade of *O. cheni* sp. nov. (96.2/94).

**Figure 6.**
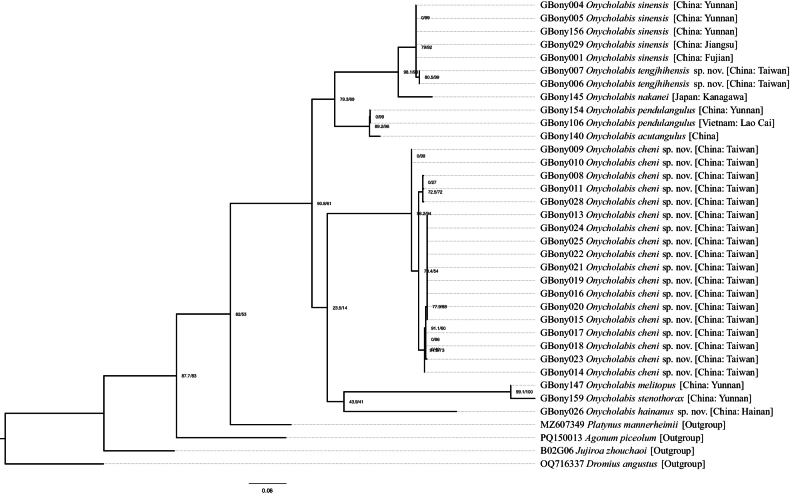
Phylogenetic trees based on the *COI* dataset from maximum-likelihood analyses; node labels provide SH-aLRT and UFBoot support values.

Crucially, the phylogenetic topology strongly supports the establishment of all three new species. *O. cheni* sp. nov. forms a well-supported independent monophyletic clade (96.2/94), and *O. hainanus* sp. nov. represents a distinct lineage on a long branch. Furthermore, *O. tengjhihensis* sp. nov. and *O. sinensis* are recovered as two separate, reciprocally monophyletic sister clades (with *O. tengjhihensis* sp. nov. supported by 80.5/99), phylogenetically establishing its validity as a distinct species (Fig. 6).

### Species delimitations

The ASAP analysis yielded multiple delimitation scenarios. The optimal partition, which received the best ASAP score of 1.0 (*p*-value = 0.021), identified ten putative species (including the four outgroups), corresponding to six OTUs within the genus *Onycholabis*. Under this optimal scenario, *O. cheni* sp. nov., *O. hainanus* sp. nov., and *O. nakanei* were successfully delimited as distinct species. However, the analysis clustered *O. sinensis* and *O. tengjhihensis* sp. nov. into a single OTU. Similarly, *O. pendulangulus* was clustered with *O. acutangulus*, and *O. melitopus* was clustered with *O. stenothorax*.

**Figure 7.**
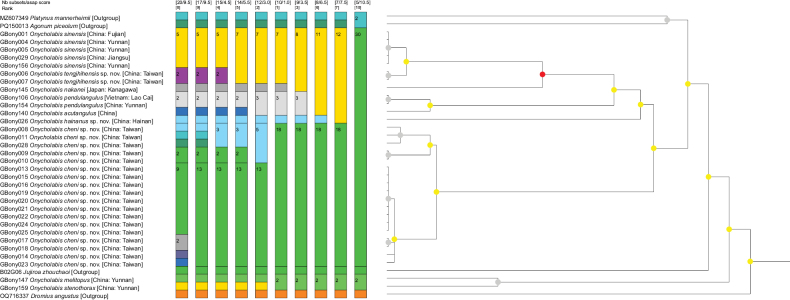
Species delimitation by ASAP analysis for the *COI* dataset. Species groupings are visualized through colored fields, each labeled with the corresponding individual count. The ASAP-score and total species richness for the dataset are displayed above the bars as the lower and upper values, respectively. The underlying cladogram provides phylogenetic support for the species boundaries delineated in the bar charts.

The second-best partition (ASAP score = 3.0, *p*-value = 0.174) proposed 12 putative species. In this scenario, *O. melitopus* and *O. stenothorax* were correctly separated into distinct species. However, the *O. cheni* sp. nov. clade was split into two distinct OTUs, while the clusters of *O. sinensis* + *O. tengjhihensis* sp. nov. and *O. pendulangulus* + *O. acutangulus* remained unresolved (Fig. 7).

**Figure 8.**
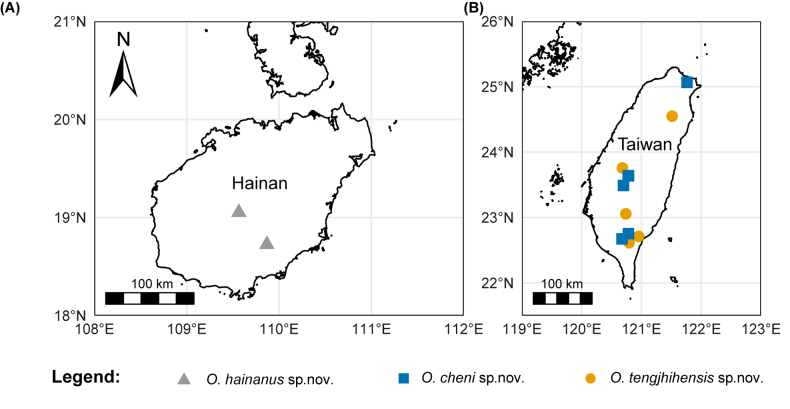
Distribution maps of the new *Onycholabis* species in **A**. Hainan and **B**. Taiwan.

**Figure 9.**
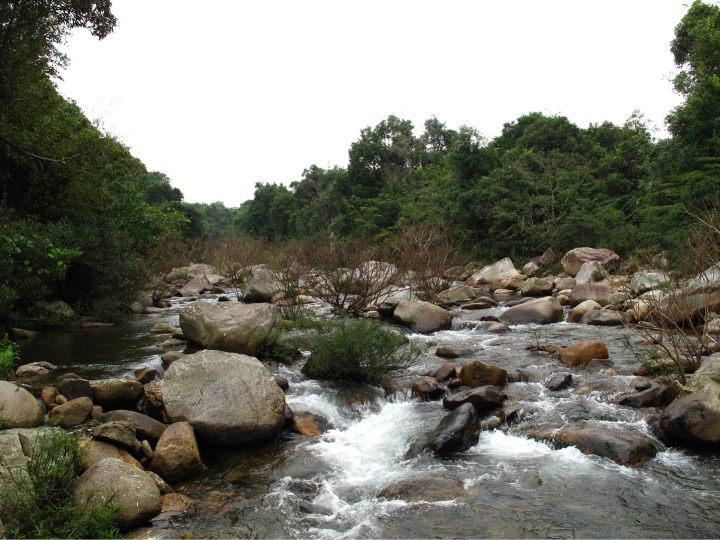
Habitat of *Onycholabis hainanus* Bolkiboev & Liang, sp. nov. (Photo by HBL).

**Figure 10.**
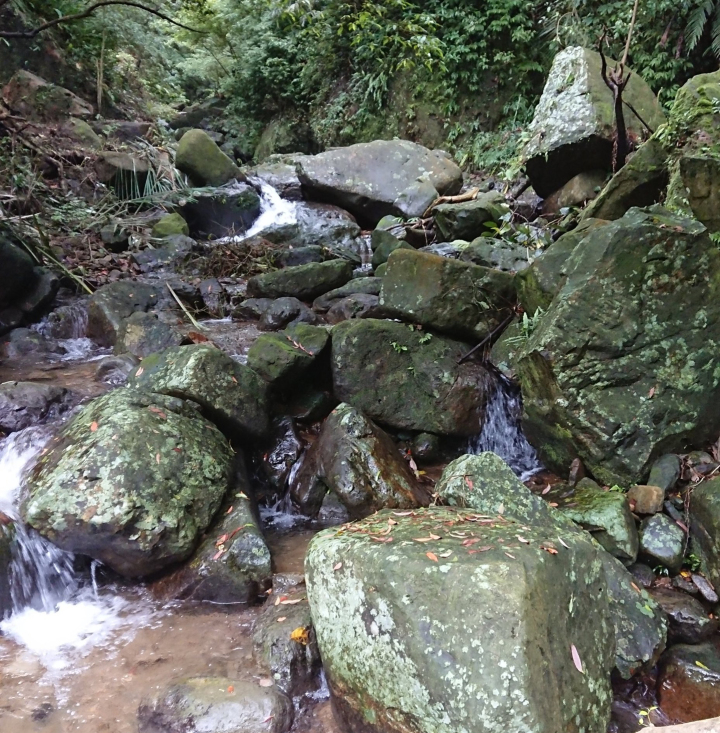
Habitat of *Onycholabis cheni* Bolkiboev & Liang, sp. nov. (Photo by S.P. Wu).

These delimitations exhibit certain inconsistencies with the topology of the phylogenetic tree. The potential reasons for these discrepancies will be further addressed in the discussion section.

### Key to species of genus *Onycholabis* Bates, 1873

(revised from Liang and Kavanaugh 2005)

**Table:** 

1	Pronotum trapezoidal, apex wide (PW/PAW=1.0–1.1), front angles angulate, sharply pointed at apex	**2**
–	Pronotum more or less cordate, apex narrow (PW/PAW=1.3–1.5), front angles round, obtuse at apex	**3**
2	Pronotum widest at apex, front angles protruded forward, then bent ventrally and sharply pointed at apex	***O. pendulangulus* Liang & Imura, 2003**
–	Pronotum widest at apical one-fifth, front angles protruded forward, sharply pointed at apex, but ventrally not bent	***O. acutangulus* Andrewes, 1923**
3	Pronotum with posterior marginal setae	**4**
–	Pronotum without posterior marginal setae	**6**
4	Eye small, tempora long (EYL/TL = 1.60–2.50)	***O. cheni* sp. nov. (Fig. 1C)**
–	Eye big, tempora short (EYL/TL = 3.0–3.56)	**5**
5	Pronotum narrower than or equal to head (PW/HW = 0.95–1.00); elytra striae strongly punctate, elytral interval 3 usually with two discal pores; sixth visible sternite 2-setose in male	***O. hainanus* sp. nov. (Fig. 1B)**
–	Pronotum wider than head (PW/HW = 1.05–1.15); elytra striae less densely punctate, elytral interval 3 usually with three discal pores; sixth visible sternite 4-setose in male	***O. sinensis* Bates, 1873**
6	Elytral apex at sutural angle acute and posteriorly projected; sixth visible sternite 2-setose in male, 4-setose in female	***O. melitopus* Bates, 1892**
–	Elytral apex at sutural angle rounded, not posteriorly projected; sixth visible sternite 2-setose or 4-setose in male, 6- to 8-setose in female	**7**
7	Sixth visible sternite with 2-setose in male, 6-setose in female; pronotum narrower relative to head (PW/HW = 0.85–0.92)	***O. stenothorax* Liang & Kavanaugh, 2005**
–	Sixth visible sternite with 4-setose in male, 8-setose in female; pronotum wider relative to head (PW/HW = 0.92–1.04)	**8**
8	Eye small, tempora long (EYL/TL = 1.77–2.08); pronotum wider than head (PW/HW=1.02–1.04)	***O. nakanei* Kasahara, 1986 (Kasahara 1986: fig. 2a)**
–	Eye big, tempora short (EYL/TL = 3.00–3.22), pronotum narrower than head (PW/HW = 0.94–0.97)	***O. tengjhihensis* sp. nov. (Fig. 1A)**

## Discussion

The present study reveals previously unrecognized species-level diversity within *Onycholabis* from southern China. The three new species described herein conform to the diagnostic concept of the genus but are clearly distinguishable from previously known congeners by stable combinations of external morphological characters and genital structures.

No specimens of *Onycholabis sinensis* Bates, 1873 were collected in Taiwan during the present study, suggesting that Kasahara may have misidentified this species when describing *Onycholabis nakanei* from Japan (Kasahara 1986: fig. 2b).

The absence of a comprehensive phylogenetic framework for Platynini complicates the assessment of relationships among species groups. Most diagnostic characters are plesiomorphic or homoplastic, leading to uncertainty in defining *Onycholabis* as a monophyletic group. Although paramere morphology, particularly the anterior hook of the left paramere, has been used to classify taxa within *Colpodes* sensu latissimo, (Schmidt 2000; Will et al. 2005). This character state is also observed in *Onycholabis*, which has contributed to its previous placement within or near the *Colpodes* complex. However, detailed examination of the aedeagal parameres, especially the structure of the apophysis of the left paramere, reveals additional differences that do not support a close relationship between *Onycholabis* and *Colpodes* sensu latissimo (Fig. 2A, C). In addition, the presence of bilaterally symmetrical testes in *Onycholabis* contrasts with the monorchid condition typical of many platynines (Will et al. 2005), supporting its distinct phylogenetic placement (Fig. 5).

We employed an integrative taxonomic approach, combining detailed morphological examination with phylogenetic analyses based on *COI* sequences to review the genus *Onycholabis*. Our results robustly support the recognition of three previously undescribed species: *O. hainanus* sp. nov., *O. tengjhihensis* sp. nov., and *O. cheni* sp. nov. Among them, *O. hainanus* sp. nov. is well supported as a distinct lineage in both morphological traits and molecular data, occupying a key systematic position within the genus.

The phylogenetic analysis recovered all *O. cheni* sp. nov. samples as a strongly supported monophyletic clade in the IQ-TREE analysis. However, we observed significant intra-specific genetic distances within this group, particularly between populations from different altitudinal gradients. Specifically, specimens collected from medium-altitude regions (Nantou, Pingtung, and Chiayi, 1000–1600 m) exhibited notable genetic divergence from those found in low-altitude areas (Keelung, 300–350 m), despite their morphological similarity. This is likely due to the pronounced geographical isolation caused by Taiwan's montane topography. Complex mountain barriers have restricted gene flow between individuals in different altitudinal habitats, thereby accelerating lineage divergence over prolonged evolutionary timescales. Such high levels of genetic diversity suggest the potential existence of topography-driven speciation within the *O. cheni* sp. nov. complex, a phenomenon also observed in alpine carabids (Sota and Vogler 2001; Weng et al. 2020).

Interestingly, our results showed notable conflicts between the distance-based ASAP delimitation and the likelihood-based IQ-TREE phylogeny. ASAP analyses struggled to correctly delimit closely related species, artificially lumping *O. sinensis* with *O. tengjhihensis* sp. nov., *O. pendulangulus* with *O. acutangulus*, and *O. melitopus* with *O. stenothorax* into single OTUs. Additionally, alternative ASAP partitions erroneously split the monophyletic *O. cheni* sp. nov. clade into multiple distinct OTUs. This discrepancy highlights the limitations of simple distance-based methods when dealing with mitochondrial *COI* sequences. On one hand, mutational saturation, particularly at third codon positions, can erode the “barcode gap”, leading to artificial clustering with near-neighbor taxa or failure to recover the monophyly of the genus. On the other hand, recent evolutionary divergence or incomplete lineage sorting can result in extremely small genetic distances between valid sister species, causing distance-based algorithms like ASAP to under-split them.

In contrast, by employing a codon-partitioning strategy and sophisticated substitution models, the ML phylogenetic inference (IQ-TREE) accounted for rate heterogeneity and multiple hits, successfully uncovering the phylogenetic signal. For instance, while *O. sinensis* is widely distributed across mainland China with low genetic variation, the endemic *O. tengjhihensis* sp. nov. from Taiwan is phylogenetically closely related to it. Despite the lack of resolution in ASAP (lumping them together due to small genetic distance), IQ-TREE successfully recovered them as reciprocally monophyletic sister clades. Given their consistent morphological diagnostic features and disjunct oceanic distribution, the recognition of *O. tengjhihensis* sp. nov. as a valid distinct species is thoroughly justified.

While this study clarifies the status of these three new species, our knowledge of evolution remains incomplete. However, relying solely on mitochondrial *COI* information is highly limited, as it represents only a single maternal locus and may not fully capture the complex evolutionary dynamics of the genus. Future studies incorporating additional nuclear markers and more extensive sampling across the East Asian archipelago and mainland China will be essential to resolve the phylogeography and potential cryptic diversity of *Onycholabis* (Schmidt et al. 2023).

## Supplementary Material

XML Treatment for Onycholabis tengjhihensis

XML Treatment for Onycholabis hainanus

XML Treatment for Onycholabis cheni

## Additional information

### Artificial Intelligence (AI) use

The authors accept full responsibility for the content of the manuscript, including the disclosure of any use of AI.

No AI tools were used in the preparation of this manuscript.

### Author ORCIDs

S. Bolkiboev https://orcid.org/0009-0004-7399-6109

S. Huang https://orcid.org/0000-0001-8357-6651

B. Kholmatov https://orcid.org/0000-0003-0003-9231

H. Liang https://orcid.org/0000-0002-9668-1167

### Data availability

The occurrence dataset generated during this study has been uploaded to the Global Biodiversity Information Facility (GBIF) and will be publicly accessible after publication. Dataset URL: https://ipt.pensoft.net/manage/resource.do?r=onycholabis_occurrence_2026#.

## Supplementary materials

Supplementary material 1 Table of localities Data type csv https://binary.pensoft.net/file/1681590This dataset is made available under the Open Database License (http://opendatacommons.org/licenses/odbl/1.0/). The Open Database License (ODbL) is a license agreement intended to allow users to freely share, modify, and use this Dataset while maintaining this same freedom for others, provided that the original source and author(s) are credited. Shokhrukh Bolkiboev, Sunbin Huang, Bakhtiyor Kholmatov, Hongbin Liang
